# Clinicopathological Profile, Stage Distribution, and Treatment Patterns of Oral Cancer at a National Referral Center in Indonesia

**DOI:** 10.3390/dj14060379

**Published:** 2026-06-18

**Authors:** Faradiba N. R. Iskandar, Vera Julia, Aulia Shifatur Rahimah, Arbi Wijaya, Bayu Brahma, Mohammad Adhitya Latief, Dwi Ariawan, Norifumi Nakamura

**Affiliations:** 1 Undergraduate Program, Faculty of Dentistry, Universitas Indonesia, Jakarta 10430, Indonesia; faradiba.naila@ui.ac.id (F.N.R.I.); aulia.shifatur61@ui.ac.id (A.S.R.); 2 Department of Oral and Maxillofacial Surgery, Faculty of Dentistry, Universitas Indonesia, Jakarta 10430, Indonesia; arbi.wijaya.t2@dc.tohoku.ac.jp (A.W.); adhityalatief@ui.ac.id (M.A.L.); dwi.ariawan02@ui.ac.id (D.A.); gigi1394nakamura@gmail.com (N.N.); 3 Dental Department, Universitas Indonesia Hospital, Depok 16424, Indonesia; 4 Division of Oral and Maxillofacial Oncology and Surgical Sciences, Graduate School of Dentistry, Tohoku University, Sendai 980-0872, Miyagi, Japan; 5 Division of Surgical Oncology, Dharmais Cancer Center Hospital, Jakarta 11420, Indonesia; bbrahma@dharmais.co.id

**Keywords:** oral cancer, late-stage presentation, oral squamous cell carcinoma (OSCC), clinicopathological profile

## Abstract

**Background**: Late-stage presentation of oral cancer remains a major challenge in low- and middle-income countries and contributes substantially to poor clinical outcomes. Data describing oral cancer presentation patterns in Indonesia remain limited. This study aimed to characterize the clinicopathological profile, stage distribution, treatment patterns, and exposure-related characteristics of oral cancer patients treated at a national referral center in Indonesia. **Methods**: A retrospective study was conducted using medical records of 404 patients with histopathologically confirmed oral malignancies treated between 2021 and 2025. Descriptive analyses were performed to summarize demographic, clinicopathological, staging, treatment-related, and exposure-related characteristics. **Results**: The mean age at diagnosis was 49.17 ± 14.11 years, with a relatively balanced sex distribution. The tongue was the most common primary tumor site (76.0%), and oral squamous cell carcinoma (OSCC) represented the predominant histopathological diagnosis (81.9%). Late-stage presentation (stage III–IV) was observed in 64.1% of all cases and increased to 70.7% among patients with available staging information, while 29.2% of patients had incomplete or undefined staging data. Surgical treatment, either alone or combined with adjuvant therapies, was the most frequently employed treatment modality. Notably, 21.5% of patients had no documented definitive oncologic treatment during the recorded treatment period. Smoking was reported by 35.4% of patients, alcohol consumption by 4.0%, and a family history of cancer by 24.8%. **Conclusions**: Advanced-stage oral cancer was highly prevalent in this referral-based cohort. The substantial burden of late-stage disease, together with incomplete staging information and the proportion of patients without documented definitive treatment, highlights challenges related to staging completeness, treatment documentation, and cancer care monitoring. These findings support efforts to strengthen early detection, referral coordination, and cancer care monitoring within the Indonesian healthcare system.

## 1. Introduction

Oral cancer remains a significant global public health challenge, contributing substantially to cancer-related morbidity and mortality worldwide. According to GLOBOCAN 2020, lip and oral cavity cancer accounts for nearly 390,000 new cases and over 180,000 deaths annually, with a disproportionate burden in Asia, which contributes the majority of global incidence and mortality [1]. In Indonesia, oral cancer represents a growing concern, with a relatively low incidence but a comparable mortality burden, suggesting suboptimal early detection and management [2]. This disparity may reflect differences in risk factor exposure, healthcare access, early detection, and treatment availability, particularly in low- and middle-income countries (LMICs).

Oral squamous cell carcinoma (OSCC) represents the predominant histopathological subtype and is strongly associated with poor prognosis, especially when diagnosed at advanced stages [3]. Although well-established risk factors, including tobacco use, alcohol consumption, and chronic irritation, play a central role in carcinogenesis, survival rates have shown limited improvement over recent decades [4]. Importantly, despite advances in diagnostic approaches, no single biomarker has demonstrated sufficient accuracy for routine clinical application, and current diagnostic tools remain limited in early detection [5,6,7]. On the other hand, prognosis is highly stage-dependent, with early detection significantly improving clinical outcomes [8]. Despite this, late-stage presentation remains highly prevalent in many low- and middle-income countries, including those in Southeast Asia, and continues to be associated with poor clinical outcomes [8,9]. Understanding the clinicopathological profile and stage distribution of oral cancer patients within referral-based healthcare settings is important for identifying patterns of disease presentation and informing future improvements in cancer care delivery. National cancer referral centers provide valuable real-world data because they receive patients from diverse geographic regions and multiple levels of healthcare. Consequently, the stage distribution observed in such settings offers important insights into the burden of advanced disease encountered in routine clinical practice.

However, most existing evidence is derived from population-based registries or studies conducted in high-income countries, which may not adequately capture real-world clinical patterns in LMIC healthcare systems. As a result, data from referral-based settings can contribute to a better understanding of clinicopathological profile, stage distribution, and treatment patterns of oral cancer in routine clinical practice [10,11,12]. In Indonesia, epidemiological and clinicopathological data on oral cancer remain limited, particularly those integrating risk exposure patterns with stage at presentation within a national referral context [13]. As the country’s primary cancer referral institution, Dharmais Cancer Hospital serves a diverse patient population and provides valuable real-world insights into disease presentation. Therefore, this study aimed to characterize the clinicopathological profile, stage distribution, treatment patterns, and exposure-related characteristics of patients with oral cancer treated at Indonesia’s national cancer referral center.

## 2. Materials and Methods

### 2.1. Study Design and Setting

This retrospective observational study was conducted at Dharmais Cancer Hospital, Indonesia’s national cancer referral center. As a tertiary referral institution, the hospital receives patients from diverse geographic regions and multiple levels of healthcare, providing a real-world representation of oral cancer cases at the endpoint of the referral pathway. Medical records of patients diagnosed with oral cancer between January 2021 and August 2025 were reviewed.

### 2.2. Study Population and Eligibility Criteria

A total sampling approach was applied. All patients with histopathologically confirmed malignant neoplasms of the oral cavity diagnosed between January 2021 and August 2025 were included in the study. Case identification was based on histopathological diagnosis and corresponding ICD-10 codes. Benign lesions and non-neoplastic conditions were excluded to ensure diagnostic specificity. Because the primary objective of this study was to describe the clinicopathological profile, stage distribution, treatment patterns, and exposure-related characteristics of oral cancer patients, all eligible oral malignancies were included regardless of histopathological subtype. A total of 404 patients met the eligibility criteria and were included in the descriptive analysis.

### 2.3. Data Collection and Variables

Data were retrospectively extracted from electronic medical records and pathology reports using a standardized data collection form. Collected variables included demographic characteristics (age and sex), clinicopathological parameters (tumor site, TNM classification, clinical stage, and histopathological diagnosis), treatment modalities, systemic comorbidities, and exposure-related variables, including smoking history, alcohol consumption, and family history of cancer. Smoking history and family history of cancer were recorded based on patient self-report documented during routine clinical assessment. Clinical staging was based on the American Joint Committee on Cancer (AJCC) 8th edition criteria when available in the medical records. For descriptive purposes, clinical stage was summarized according to individual stages and grouped as early-stage (stage I–II) and late-stage (stage III–IV) disease. Given the retrospective nature of the study, data completeness depended on the quality of routine clinical documentation. Missing or incomplete information was observed predominantly in TNM classification and staging-related variables. Detailed exposure-related information, including smoking duration and smoking intensity, was not consistently available across records and therefore could not be analyzed further.

### 2.4. Statistical Analysis

Descriptive statistics are used to summarize demographic characteristics, clinicopathological findings, stage distribution, treatment patterns, comorbidities, and exposure-related variables among all included oral malignancies. Categorical variables are presented as frequencies and percentages, whereas continuous variables are summarized using means with standard deviations or medians with interquartile ranges, as appropriate. Clinical stage is described both according to individual AJCC stages and as grouped categories of early-stage (stage I–II) and late-stage (stage III–IV) disease. Cases with incomplete or undefined staging information were reported separately and are not included in stage-specific summaries. Given the retrospective nature of the study, missing data were not imputed and are reported as observed. All statistical analyses were conducted using IBM SPSS Statistics version 30.0 (IBM Corp., Armonk, NY, USA) and R software version 4.6.0 (R Foundation for Statistical Computing, Vienna, Austria) in RStudio (Posit Software, PBC, Boston, MA, USA). The study was reported in accordance with the STROBE guidelines for observational studies.

### 2.5. Ethical Consideration

This study was conducted in accordance with the Declaration of Helsinki and approved by the Research Ethics Committee of Dharmais Cancer Hospital, West Jakarta, Indonesia (Approval No. DP.04.03/11.10/179/2025). The requirement for informed consent was waived due to the retrospective nature of the study and the use of anonymized secondary data. Patient confidentiality was maintained throughout the study.

## 3. Results

### 3.1. Patient Characteristics

**Figure 1:**
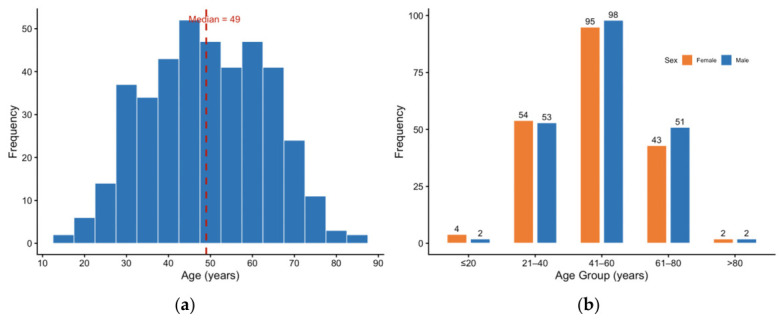
Age distribution of the study population. (**a**) Histogram showing the distribution of patient age; (**b**) Age group distribution stratified by sex.

A total of 404 patients were included in this study. The study population showed a relatively balanced sex distribution with 206 males (51.0%) and 198 females (49.0%). The mean age at diagnosis was 49.17 ± 14.11 years (range: 14–86 years), indicating a predominance of middle-aged and older individuals (Table 1 and Figure 1). 

**Figure 2:**
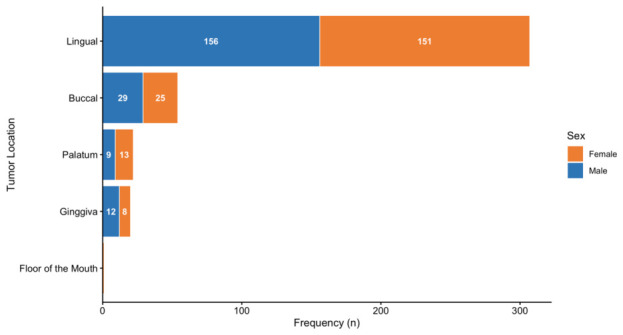
Distribution of primary tumor location stratified by sex. The tongue (lingual) was the most common tumor site in both males and females, followed by the buccal mucosa.

The predominant ethnic groups in the cohort were Betawi (29.2%), Javanese (27.7%), and Sundanese (21.5%) (Table S1). The tongue was the most common primary tumor site (76.0%), followed by the buccal mucosa (13.4%), palate (5.4%), and gingiva (5.0%) (Figure 2). Histopathologically, OSCC was the predominant diagnosis (81.9%), with other malignancies observed in smaller proportions (Table 2). Regarding systemic conditions, hypertension (21.8%) and diabetes mellitus (12.9%) were the most frequently reported comorbidities, while most patients had no documented systemic disease (Table 3).

### 3.2. Stage Distribution

**Figure 3:**
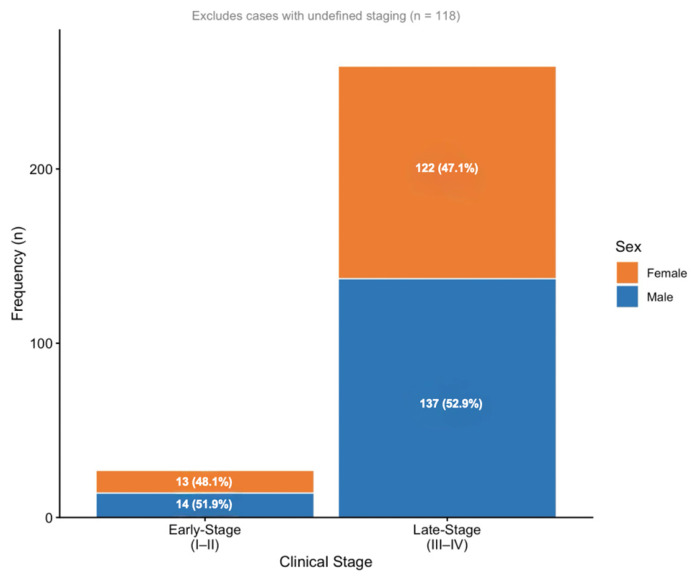
Distribution of clinical stages, divided into early and late stages, based on sex.

The distribution of tumor stage demonstrated a marked predominance of advanced disease at presentation. Stage IV was the most common stage (52.5%), followed by stage III (11.6%), whereas early-stage disease was relatively uncommon, with stage II accounting for 5.4% and stage I for only 1.2% of cases (Table S5). Among all included cases, late-stage presentation (stage III–IV) accounted for 64.1% of patients, while early-stage disease (stage I–II) represented 6.7% (Figure 3). The remaining 29.2% had incomplete or undefined staging data. When the analysis was restricted to cases with defined staging information, the proportion of late-stage disease increased to 70.7%, further emphasizing the predominance of advanced-stage presentation. Incomplete classification was also observed across TNM components, including Tx (30.2%), Nx (33.2%), and Mx (49.3%), which may affect the precision of stage distribution estimates (Tables S2–S4). Among patients with available TNM data, advanced tumor burden was reflected by a high proportion of T3–T4 lesions and nodal involvement (N1–N2), whereas confirmed distant metastasis (M1) was less frequent (7.4%) (Tables S2–S4). To illustrate the potential impact of missing staging information on stage distribution estimates, an exploratory scenario analysis was performed in which all unstaged cases were assumed to represent late-stage disease. Under this hypothetical scenario, the proportion of late-stage presentation increased from 64.1% (259/404) to 93.3% (377/404) of the total cohort. This assumption substantially increased the estimated prevalence of late-stage disease. However, because the true stage distribution among unstaged cases is unknown, these estimates should be interpreted with caution.

### 3.3. Treatment Patterns

Treatment patterns varied across the cohort but were largely consistent with the predominance of advanced-stage disease. Surgical management was the most frequently employed modality, either as a single treatment (23.0%) or in combination with chemotherapy (18.8%) and/or radiotherapy (12.6%). Multimodal treatment involving surgery combined with both chemotherapy and radiotherapy was observed in 8.4% of patients. Non-surgical approaches were less common, including chemotherapy alone (7.4%), radiotherapy alone (1.2%), and chemoradiotherapy (1.5%). Notably, a considerable proportion of patients did not receive definitive oncologic treatment (21.5%) (Table 4). This group included patients who underwent diagnostic biopsy without subsequent treatment as well as those who did not receive definitive oncologic treatment during the recorded treatment period. These findings indicate variability in treatment delivery and may reflect incomplete treatment documentation or limitations in treatment records available within the institutional database.

### 3.4. Exposure-Related Characteristics

**Figure 4:**
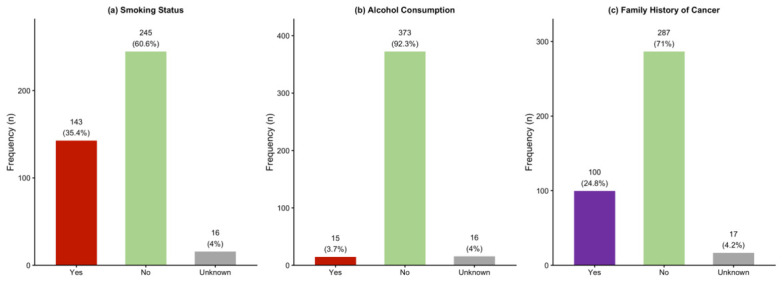
Distribution of exposure-related characteristics among patients. (**a**) Smoking status distribution; (**b**) Alcohol consumption patterns; (**c**) Family history of cancer.

The distribution of exposure-related characteristics was heterogeneous. Most patients had no history of smoking (60.6%), while 35.4% reported smoking, and 4.0% were classified as unknown. Alcohol consumption was relatively uncommon (4.0%), with the majority reporting no alcohol use (92.3%). A family history of cancer was present in 24.8% of patients. Overall, no single exposure-related characteristic was consistently observed across the cohort (Figure 4).

## 4. Discussion

This study demonstrates a substantial burden of late-stage oral cancer presentation in a national referral center in Indonesia, with late-stage disease observed in 64.1% of patients and increasing to 70.7% among cases with complete staging. This pattern is consistent with reports from other LMICs, where advanced-stage presentation remains highly prevalent and is associated with poor clinical outcomes [9,11]. The demographic and clinicopathological characteristics observed in this cohort are broadly consistent with existing literature. The predominance of middle-aged and older patients is consistent with the cumulative nature of carcinogenesis [14,15], while the slightly higher proportion of male patients is consistent with previous studies reporting greater exposure to behavioral risk factors among men [16,17,18]. The tongue was identified as the most common tumor site in this cohort, consistent with previous reports identifying the tongue as a common site for oral squamous cell carcinoma [17]. However, the predominance of tongue lesions observed in this study appears higher than that reported in several Asian cohorts, where buccal mucosa lesions are also frequently encountered, particularly in populations with widespread betel quid and smokeless tobacco use. A recent systematic review of oral cancer epidemiology in Asia demonstrated substantial regional variability in tumor site distribution, with tongue cancer predominating in several East and Southeast Asian populations, while buccal mucosa lesions were more common in regions with different behavioral risk exposures [19].

The high proportion of tongue cancer observed in the present cohort should be interpreted cautiously. Although tongue cancer has been reported as a common anatomical site in several Asian populations, the proportion observed in this study appears higher than that reported in many regional cohorts. Because this study was conducted in a single national referral center, the observed anatomical distribution may reflect an institutional case-mix, regional demographic characteristics, referral patterns, or other unmeasured factors. However, the present dataset does not permit determination of the underlying reasons for this distribution. Despite the anatomical accessibility of tongue lesions, advanced-stage presentation remained highly prevalent, emphasizing the continued importance of early detection strategies and prompt clinical evaluation of suspicious oral lesions [20]. Nevertheless, these interpretations should be considered exploratory, as several factors that may influence stage at presentation were not directly measured in this study. The findings should therefore be interpreted cautiously and may reflect broader challenges related to oral cancer detection, documentation, and care delivery within referral-based settings. The predominance of late-stage presentation observed in this study is consistent with reports from several Southeast Asian countries, where advanced-stage oral cancer remains highly prevalent at initial diagnosis. Studies from Thailand, Malaysia, Vietnam, and the Philippines have also reported that most patients present with stage III–IV disease, generally ranging from approximately 60% to 80% of cases [21,22,23,24]. This regional pattern highlights the persistent burden of advanced-stage oral cancer across Southeast Asia and underscores the need for continued efforts to promote earlier recognition and management of suspicious oral lesions. Nevertheless, the persistence of advanced-stage disease despite the anatomical accessibility of oral lesions underscores the need for more effective early detection strategies, improved clinical documentation, and strengthened coordination of oral cancer care across the region.

A key finding of this study is the substantial proportion of incomplete staging data, particularly in TNM classification and overall clinical stage. This may reflect limitations in retrospective clinical documentation and data completeness within the referral setting. While this may partly reflect the limitations of retrospective data collection, it also highlights challenges related to the completeness and consistency of routine clinical documentation. Incomplete staging information may limit the interpretation of epidemiological findings and reduce the completeness of clinical records used for patient management. To illustrate the potential impact of missing staging information on stage distribution estimates, an exploratory scenario analysis was performed in which all unstaged cases were assumed to represent late-stage disease. Under this hypothetical scenario, the proportion of late-stage presentation increased from 64.1% to 93.3% of the total cohort. Although this assumption substantially increased the estimated prevalence of advanced-stage disease, the overall pattern of late-stage predominance remained unchanged. Because the mechanism underlying the missing staging data could not be determined retrospectively, the possibility of bias cannot be excluded. Nevertheless, the high proportion of missing staging data warrants cautious interpretation of stage-related estimates.

Treatment patterns in this cohort largely reflect the predominance of advanced-stage disease, with frequent use of surgical and multimodal approaches [25]. However, a notable proportion of patients did not receive definitive treatment or underwent only a diagnostic biopsy. From a clinical perspective, this finding is important because the absence of documented definitive treatment following histopathological diagnosis may represent a gap in treatment documentation or continuity of care. Because linkage with external referral facilities, regional cancer registries, and follow-up records was unavailable, the subsequent treatment trajectories of these patients could not be verified. Consequently, it remains unclear whether these patients subsequently received treatment elsewhere, experienced treatment abandonment, declined treatment, or were lost during referral transitions. Therefore, the absence of documented treatment should not be interpreted as definitive treatment abandonment. Similar challenges in documenting and tracking treatment after diagnosis have been reported in other low- and middle-income settings. Although the underlying causes could not be directly evaluated in this study, the findings highlight the need for future investigations incorporating referral-network audits and registry linkage to better characterize barriers to treatment completion after diagnosis [11,26].

Previous studies have highlighted the potential influence of patient-, provider-, and system-level factors on oral cancer presentation. Reported factors include limited awareness of early symptoms, delayed healthcare-seeking behavior, restricted access to specialist services, referral inefficiencies, and broader healthcare delivery challenges [27,28,29,30]. These factors have been described within conceptual frameworks of diagnostic and treatment delay and are frequently discussed in the oral cancer literature as potential contributors to advanced-stage presentation. However, these variables were not directly measured in the present study and therefore cannot be used to explain the stage distribution observed in this cohort. Consequently, any interpretation regarding the contribution of patient-, provider-, or system-level delays should be considered speculative and hypothesis-generating rather than confirmatory. The predominance of advanced-stage disease observed in this study should instead be interpreted primarily as a descriptive finding within this referral-based cohort. Nevertheless, the persistence of advanced-stage presentation highlights the need for future research incorporating healthcare pathway variables, referral-network assessments, patient-level access indicators, and measures of healthcare utilization. Such approaches may provide a more comprehensive understanding of factors associated with oral cancer presentation in Indonesia and help inform strategies aimed at improving earlier diagnosis and care delivery [31,32,33].

This study has several limitations. First, the retrospective single-center design may limit the generalizability of the findings, particularly because Dharmais Cancer Hospital functions as a national tertiary referral center that may receive a disproportionate number of advanced or complex cases. Second, several potentially relevant variables, including symptom duration, socioeconomic status, healthcare accessibility, and treatment-related barriers, were unavailable in the medical records and therefore could not be evaluated directly. Consequently, factors potentially associated with stage at presentation could not be comprehensively assessed. Third, a substantial proportion of patients had incomplete or undefined staging information, particularly within TNM classification and overall clinical stage. Because the mechanism underlying the missing staging data could not be determined retrospectively, the possibility of bias cannot be excluded, and stage-related estimates should be interpreted with caution. Although an exploratory scenario analysis demonstrated that the predominance of late-stage disease remained unchanged under alternative assumptions, incomplete staging information remains an important limitation of the dataset. Fourth, linkage with external referral facilities, regional cancer registries, and longitudinal follow-up records was unavailable. Consequently, the subsequent treatment status and clinical outcomes of patients who did not receive documented definitive oncologic treatment within the institutional records could not be verified. It therefore remains uncertain whether these patients received treatment elsewhere, declined treatment, experienced treatment abandonment, or were lost during referral transitions. As a result, the absence of documented treatment should not be interpreted as definitive treatment abandonment. Finally, the disproportionately high proportion of tongue lesions observed in this cohort may partly reflect referral patterns, institutional case-mix characteristics, or other unmeasured factors rather than the anatomical distribution of oral cancer in the broader Indonesian population. Therefore, these findings should not be considered nationally representative. Despite these limitations, this study provides important real-world data regarding oral cancer presentation patterns at Indonesia’s national cancer referral center. The findings highlight the substantial burden of advanced-stage disease, challenges related to staging completeness and treatment documentation, and the need for improved clinical documentation, cancer care monitoring, and multicenter investigations to better characterize oral cancer presentation across diverse healthcare settings in Indonesia.

## 5. Conclusions

This study provides a descriptive overview of oral cancer presentation at Indonesia’s national cancer referral center. Advanced-stage disease predominated within the cohort, with tongue cancer representing the most common tumor site. A substantial proportion of patients had incomplete staging information, and a notable number lacked documented definitive oncologic treatment, highlighting challenges related to clinical documentation and treatment tracking in routine practice. Although the findings should be interpreted in light of the study’s retrospective design and referral-based setting, they offer important real-world insights into oral cancer presentation patterns in Indonesia. These findings support continued efforts to strengthen early detection initiatives, improve clinical documentation and cancer care monitoring, and expand multicenter research to better characterize oral cancer burden across diverse healthcare settings in Indonesia.

## Figures and Tables

**Table 1:** Summary statistics of patient age.

Category	Result
Mean	49.17
Median	49.00
Mode	47
Minimum	14
Maximum	86
Std. Deviation	14.11
Range	72
IQR	22

**Table 2:** Distribution of histopathological diagnoses.

Histopathological Diagnosis	Frequency (*n*)	Percentage (%)
Adenocarcinoma	4	1.0
Adenoid Cystic Carcinoma	8	2.0
Adenosquamous Cell Carcinoma	1	0.2
Carcinoma ex Pleomorphic Adenoma	1	0.2
Chondrosarcoma	1	0.2
Clear Cell Carcinoma	1	0.2
Epithelioid-Hemangioendothelioma	1	0.2
Ewing Sarcoma	1	0.2
Fibrosarcoma	1	0.2
Large B-Cell Lymphoma	1	0.2
Malignant Fibrous Histiocytoma	1	0.2
Melanoma	3	0.7
Mucoepidermoid Carcinoma	10	2.5
Myoepithelial Carcinoma	1	0.2
Non-Hodgkin Lymphoma	5	1.2
Oral Squamous Cell Carcinoma (OSCC)	331	81.9
Papillary Squamous Cell Carcinoma	1	0.2
Sarcoma	1	0.2
Small-cell Osteosarcoma	1	0.2
Spindle-cell Rhabdomyosarcoma	1	0.2
Undifferentiated Carcinoma of the Palate	1	0.2
Unspecified Carcinoma	28	6.9

**Table 3:** Distribution of systemic comorbidities.

Systemic Condition	Frequency (*n*)	Percentage (%)
Hypertension	88	21.8
Hypotension	7	1.7
Diabetes Mellitus	52	12.9
Tuberculosis	14	3.4
Pneumonia	8	1.9
Anemia	8	1.9
Lung Disease	1	0.2
Kidney Disease	9	2.2
Heart Disease	8	1.9
Stroke	5	1.2
Others	13	3.1
None	250	61.9

**Table 4:** Distribution of treatment history.

Treatment History	Frequency (*n*)	Percentage (%)
Chemoradiotherapy	6	1.5
Chemoradiotherapy + Chemotherapy	2	0.5
Chemotherapy	30	7.4
Chemotherapy + Radiotherapy	5	1.2
Radiotherapy	5	1.2
Surgery	93	23.0
Surgery + Chemoradiotherapy	10	2.5
Surgery + Chemotherapy	76	18.8
Surgery + Chemotherapy + Radiotherapy	34	8.4
Surgery + Radiotherapy	51	12.6
No definitive oncologic treatment	87	21.5
Unavailable	5	1.2
Total	404	100

Percentages may not total exactly 100% due to rounding to one decimal place.

## Data Availability

The data are not publicly available due to privacy and ethical restrictions. The data that support the findings of this study are available from the corresponding author upon reasonable request.

## Supplementary Materials

The following supporting information can be downloaded at https://www.mdpi.com/article/10.3390/dj14060379/s1, Table S1: Distribution of patient ethnicity; Table S2: Distribution of tumor (T) stage; Table S3: Distribution of nodal (N) stage; Table S4: Distribution of metastasis (M) stage; Table S5: Distribution of clinical stage.

## Patents

This study did not result in any patents.

## Abbreviations

The following abbreviations are used in this manuscript:

LMICs	Low- and middle-income countries
OSCC	Oral squamous cell carcinoma
TNM	Tumor Nodes Metastasis
