# Supplementary material for: Clinicopathological Profile, Stage Distribution, and Treatment Patterns of Oral Cancer at a National Referral Center in Indonesia
Source: Dent J (Basel). 2026 Jun 18;14(6):379. doi: 10.3390/dj14060379 (PMC13297957; doi:10.3390/dj14060379)
Supplement: Supplementary file 1 [file dentistry-14-00379-s001.zip › dentistry-4259687-supplementary.pdf]

Supplementary Materials

Table S1. Distribution of patient ethnicity.

Ethnicity	Frequency (n)	Percentage (%)
Aceh	4	1.0
Ambon	1	0.2
Batak	16	4.0
Bengkulu	3	0.7
Betawi	118	29.2
Bugis	2	0.5
Tionghoa (Chinese)	13	3.2
Dayak	1	0.2
Jambi	3	0.7
Jawa (Javanese)	112	27.7
Lampung	2	0.5
Makassar	2	0.5
Maluku (Moluccan)	2	0.5
Manado	1	0.2
Melayu (Malay)	10	2.5
Nias	2	0.5
NTB (West Nusa Tenggara)	1	0.2
NTT (East Nusa Tenggara)	2	0.5
Padang (Minangkabau)	10	2.5
Palembang	4	1.0
Papua (Papuan)	6	1.5
Sunda (Sundanese)	87	21.5
Ternate	1	0.2
Unknown	1	0.2
**Total**	404	100.0

Table S2. Distribution of tumor (T) stage.

T Stage	Frequency (n)	Percentage (%)
T1	10	2.5
T2	49	12.1
T3	55	13.6
T4	167	41.3
Tx (Undefined)	122	30.2
Unknown	1	0.2
**Total**	**404**	**100.0**

Table S3. Distribution of nodal (N) stage.

N Stage	Frequency (n)	Percentage (%)
N0	46	11.4
N1	113	28.0
N2	74	18.3
N3	36	8.9
Nx (Undefined)	134	33.2
Unknown	1	0.2
**Total**	**404**	**100.0**

Table S4. Distribution of metastasis (M) stage.

M Stage	Frequency (n)	Percentage (%)
M0	174	43.1
M1	30	7.4
Mx (Undefined)	199	49.3
Unknown	1	0.2
**Total**	404	100.0

Table S5. Distribution of clinical stage (stage I–IV).

Clinical Stage	Frequency (n)	Percentage (%)
Stage I	5	1.2
Stage II	22	5.4
Stage III	47	11.6
Stage IV	212	52.5
Undefined	118	29.2
**Total**	**404**	**100.0**

Note: Substages (including T4a–T4b, N2a–N2c, N3a–N3c, and clinical Stage IVA–IVC) were combined into their corresponding main stage categories for clarity.
